# The molecular regulation of oligodendrocyte development and CNS myelination by ECM proteins

**DOI:** 10.3389/fcell.2022.952135

**Published:** 2022-09-06

**Authors:** Momona Yamada, Miho Iwase, Binri Sasaki, Nobuharu Suzuki

**Affiliations:** Department of Molecular and Cellular Biology, Graduate School of Medical and Dental Sciences, Tokyo Medical and Dental University (TMDU), Tokyo, Japan

**Keywords:** myelin, oligodendrocyte, extracellular matrix, laminin, tenascin, fibronectin, hyaluronan, chondroitin sulfate proteoglycan

## Abstract

Oligodendrocytes are myelin-forming cells in the central nervous system (CNS). The development of oligodendrocytes is regulated by a large number of molecules, including extracellular matrix (ECM) proteins that are relatively less characterized. Here, we review the molecular functions of the major ECM proteins in oligodendrocyte development and pathology. Among the ECM proteins, laminins are positive regulators in oligodendrocyte survival, differentiation, and/or myelination in the CNS. Conversely, fibronectin, tenascin-C, hyaluronan, and chondroitin sulfate proteoglycans suppress the differentiation and myelination. Tenascin-R shows either positive or negative functions in these activities. In addition, the extracellular domain of the transmembrane protein teneurin-4, which possesses the sequence homology with tenascins, promotes the differentiation of oligodendrocytes. The activities of these ECM proteins are exerted through binding to the cellular receptors and co-receptors, such as integrins and growth factor receptors, which induces the signaling to form the elaborated and functional structure of myelin. Further, the ECM proteins dynamically change their structures and functions at the pathological conditions as multiple sclerosis. The ECM proteins are a critical player to serve as a component of the microenvironment for oligodendrocytes in their development and pathology.

## Introduction

In the central nervous system (CNS), oligodendrocytes (OLs) form myelin that is required for the rapid propagation of action potential and for the maintenance of axonal homeostasis. During the development at the embryonic stages, OL precursor cells (OPCs) are generated from neural stem cells in the subventricular zone (SVZ), and then, they proliferate and migrate to the appropriate regions in the white matter. At the postnatal stage, OPCs initiate to differentiate into OLs and subsequently myelinate adjacent axons (Baumann and Pham-Dinh, 2001). Since the roles of OLs are crucial for proper functioning of the CNS, defects in their functions cause demyelinating and dysmyelinating disorders, such as multiple sclerosis (MS) and leukodystrophy. Each process of OL development is orchestrally and precisely regulated by numerous molecules, such as growth factors/cytokines and their receptors, cell adhesion molecules, cytoplasmic adaptor proteins and kinases, transcription factors, and myelin proteins (Elbaz and Popko, 2019). In addition to these molecules, extracellular matrix (ECM) proteins are critically involved in OL development, since OLs are exposed to ECM on a number of occasions during the processes. Moreover, ECM proteins act as a key player at the pathological conditions of MS. In this review, we focus on the characterization of ECM proteins, laminins (the basement membrane- and non-basement membrane-types), tenascins, fibronectin (the cellular- and plasma-types), hyaluronan, chondroitin sulfate proteoglycans (CSPGs), and a transmembrane protein teneurin-4, which possesses the sequence homology with tenascins and possibly functions as an ECM protein, in OL development and myelination. We further discuss about the molecular mechanisms of their functions in both normal and pathological tissue conditions.

## The functions of ECM proteins in OL development and pathology in the CNS

### Laminins

Laminins, cell adhesive glycoproteins in basement membrane, are a heterotrimeric protein that is composed of α, β, and γ chains. Five α chains (α1-5), four β chains (β1-4), and three γ chains (γ1-3) have been identified and various combinations of the 3 chains produce the isoforms of laminins: for instance, laminin-111 consists of α1, β1, and γ1 chains (Yao, 2017). The 5 tandem laminin G-domain-like (LG) modules (LG1-5) are located in the C-terminal region of α chain and possess binding activities for cellular receptors. The LG1-3 and LG4-5 modules bind to integrins and α-dystroglycan/sulfatide/heparan sulfate proteoglycans (HSPGs), respectively (Suzuki et al., 2005). Laminins regulate survival, migration, proliferation, differentiation, and myelination of OL lineage cells through the receptors (Kang and Yao, 2022). The gene mutations of laminin α2 chain in mice cause the defects in OPC survival and delayed myelination (Relucio et al., 2012) and those in human are linked to the abnormality in the white matter (Arreguin and Colognato, 2020), indicating that laminin is required for proper myelination in the CNS. Further, not only congenital defects in the laminin function but also the involvement of laminin activities in pathogenesis of stroke and MS are reported (Kang and Yao, 2022).

In the CNS, the expression of both the basement membrane type and the non-basement membrane type of laminins has been observed. In the vascular basement membrane, laminin α1, α2, α4, and α5 chains, but not the α3 chain, are expressed in the postnatal mouse brain (Suzuki et al., 2019). As the non-basement membrane type, laminin α2 chain is expressed along axons from the brain stem to the spinal cord (Colognato et al., 2002). In addition, the expression of laminin γ1 chain, which is a component of most of laminin isoforms, is detected in the pericellular regions of radial glial cells and OPCs in the SVZ (Relucio et al., 2012). Further, laminin is also found in the CNS parenchyma (Lau et al., 2013). The main function of the non-basement membrane laminins is the promotive effect on survival and morphogenesis of OL lineage cells and myelination, while the basement membrane type is active on migration and survival of OPCs, as discussed below.

**FIGURE 1:**
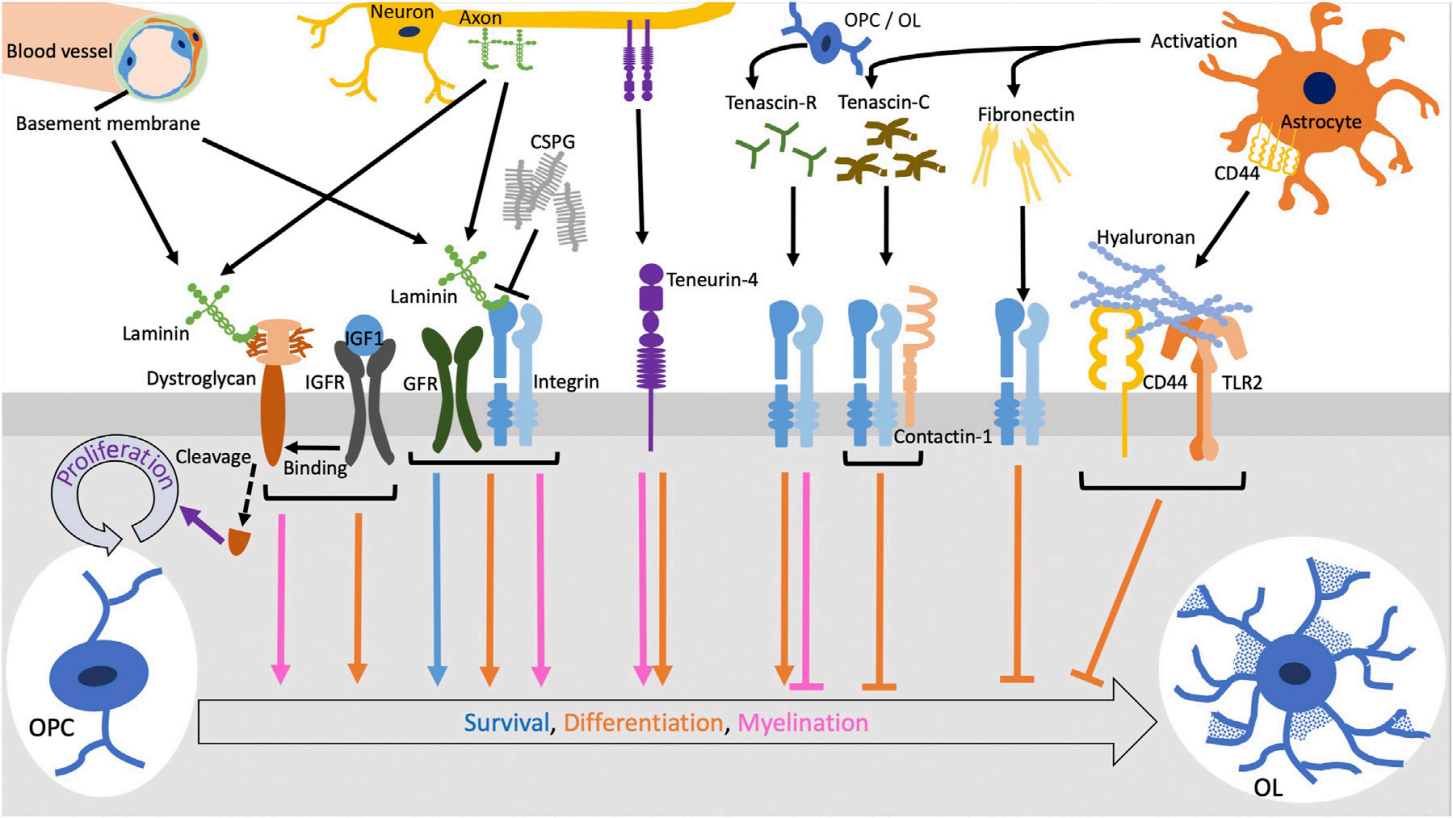
The functions of ECM molecules through cellular receptors. Laminins expressed in vascular basement membrane or along axons promote survival and differentiation of oligodendrocytes (OLs) and myelination via integrins in coordination with growth factor receptors (GFRs). Chondroitin sulfate proteoglycans (CSPGs) form aggregates and inhibit the laminin-integrin signaling. The cleavage of dystroglycan downstream of the binding to laminin induce OL precursor cell (OPC) proliferation. OL differentiation and myelination by the laminin-dystroglycan binding is enhanced when dystroglycan interacts with the complex of insulin-like growth factor (IGF1) and IGF receptor (IGFR). Tenascin-R and teneurin-4 promotes differentiation of OLs while tenascin-C inhibits it via the interaction of integrins with contactin-1. Fibronectin aggregation secreted by activated astrocytes negatively regulates OL differentiation. Also, hyaluronan inhibits OL differentiation through the TLR2 signaling.

Integrin α6β1 is the most characterized receptor for laminins. The study using CNS tissues and primary OL lineage cells from knockout mice of integrin α6 demonstrated the reduced survival of OLs, particularly when they were cultured on laminin-211 (Colognato et al., 2002). However, the phenotypes of mutant mice that are gene-manipulated to decrease the expression or activity of integrin β1 are mild, while integrin β1 promotes the formation of myelin membrane on laminin-211 in culture (Buttery and ffrench-Constant, 1999; Benninger et al., 2006; Câmara et al., 2009). Moreover, there are several evidence that show the positive function of laminin-211, which is expressed on neuronal axons, in OL survival and differentiation by the synergetic effects of integrin α6β1 with soluble growth factors/their receptors and the other cell surface molecules (Figure 1). Binding of laminin-211 to integrin α6β1 enhances OL survival and differentiation through the activation of tyrosine kinase Fyn and the mitogen-activated protein kinase (MAPK) pathway, cooperatively with the signaling of the soluble form of neuregulin-1 (Colognato et al., 2004). Sulfatide, a sulfated molecule of galactosylceramide (GalCer), is one of the major components to form membrane microdomains of OLs and positively regulates myelin basic protein (MBP) expression and myelination. Sulfatide is co-localized with integrin α6β1 in lipid rafts when the OPC line OLN-GS that overexpresses GalCer and sulfatide is cultured on laminin-211. The interaction between integrin α6β1 and sulfatide in the membrane microdomains is crucial for the positive function of laminin-211 in OL differentiation and myelin formation (Baron et al., 2014). Further, the activities of the integrin binding domain E8, which is composed of the C-terminal region of the heterotrimeric rod domain of the 3 chains (αβγ) and LG1-3 modules in α chain, from the laminin isoforms expressed in the vascular basement membrane of the CNS were recently accessed. Laminin-211E8, -411E8, and-511E8 exhibited migration activity of OPCs, whereas laminin-411E8 and -511E8 protected a higher number of OPCs from apoptosis (Suzuki et al., 2019).

Dystroglycan is a non-integrin type of the receptors for laminins. There are two forms α- and β-dystroglycan, which are encoded in the same gene and post-translationally cleaved. α-dystroglycan, a highly glycosylated cell surface protein that is associated with β-dystroglycan, a transmembrane protein, binds to laminins through its oligosaccharides (Hohenester, 2018). Both the blocking antibody against dystroglycan and knockdown of dystroglycan reduce the morphological maturation, but not the expression of MBP, in OLs (Colognato et al., 2007). The interaction of dystroglycan with laminin-211 promotes the growth of OL processes due to the activation of the filopodia and branching formation. Focal adhesion kinase (FAK) is activated in the tips of filopodia and enhances the process dynamics in the early OL differentiation stage (Eyermann et al., 2012). In addition, insulin-like growth factor-1 (IGF-1) regulates the interaction between laminin-211 and dystroglycan via the activation of the MAPK pathway (Galvin et al., 2010) (Figure 1). Furthermore, the intracellular domain of β-dystroglycan is cleaved by metalloproteinases (MMPs), which promotes OPC proliferation on laminin-211 (Leiton et al., 2015) (Figure 1). Recently, a cross-talk signaling between integrin α6β1 and dystroglycan has been reported. The receptor-like protein tyrosine phosphatase α (PTPα) is essential for MBP expression and cell spreading during laminin-211-induced oligodendrocyte differentiation. PTPα complexes with integrin α6β1 and dystroglycan to transduce Fyn activation upon laminin-211 engagement. In this way, PTPα mediates a subset of laminin-211-induced signals required for differentiation, including mTOR-dependent Akt activation but not Erk1/2 activation (Ly et al., 2018). Interestingly, laminin α chain LG4-5 modules, which bind to α-dystroglycan, are often cleaved and modulate their signaling to coordinate with the integrin signaling by the main part of laminins in other tissues (Utani et al., 2001; Smirnov et al., 2002; Ido et al., 2004; Schéele et al., 2005; Yamashita et al., 2008; Suzuki et al., 2010). This type of proteolysis probably occurs in the CNS tissue as well. Therefore, laminins exert their appropriate activities via the receptor interactions, depending on the developmental or pathological status of OLs.

### Tenascins

Tenascins are multimeric ECM glycoproteins. There are four tenascin family members in vertebrates, tenascin-C, -R, -W, and -X. Tenascins consist of the common domain structure, which is composed of heptad repeats, epidermal growth factor (EGF)-like repeats, fibronectin-type III repeats, and fibrinogen-like globe (Chiquet-Ehrismann, 2004). Tenascins bind to cell surface integrins and non-integrin types of receptors, including Toll-like receptor 4 (TLR4), and to ECM proteins, such as versican, brevican, and lectican, in order to exert their functions (Aspberg et al., 1997; Church et al., 2016; Haage et al., 2019). The expression pattern of each tenascin is tissue- and/or developmental stage-specific. During the development of mouse brain, tenascin-C and -R are expressed at early and late postnatal stages, respectively (Czopka et al., 2009). In culture, OPCs dominantly express tenascin-C and OLs abundantly express tenascin-R, which is consistent with the *in vivo* expression pattern (Czopka et al., 2009; Zhao et al., 2009). The expression of tenascin-C is also found from reactive astrocytes in the pathological condition with demyelination. In contrast, tenascin-R is expressed from OPCs recruited to the lesion tissue (Zhao et al., 2009).

The two CNS tenascins, -C and -R, show different functions in the differentiation from OPCs to OLs. Tenascin-R exhibits positive roles in OL differentiation, except for the remyelination process as discussed later. Differentiating OLs, positive for anti-sulfatide antibody O4, adhere to tenascin-R and increase the expression of MBP (Pesheva et al., 1997; Czopka et al., 2009). The autocrine effect of tenascin-R expression and the requirement of sulfatide are observed in its positive regulation for OL differentiation (Pesheva et al., 1997; Czopka et al., 2009). The ablation of tenascin-R gene expression results in the reduction of OL differentiation and the retardation of MBP expression (Czopka et al., 2009). In contrast, tenascin-C negatively regulates the differentiation of OLs. OLs cultured on tenascin-C express the lower level of MBP (Czopka et al., 2009; Czopka et al., 2010). The binding of tenascin-C to contactin-1 in the lipid rafts of OL plasma membrane recruits Fyn, a key regulator of OL differentiation, to the scaffolding complex in the cytoplasmic region (Figure 1). This molecular interaction upregulates the phosphorylation of Tyr531 in Fyn, which inactivates itself. Downstream of this signaling, the expression of Sam68 necessary for OL differentiation and MBP expression is decreased (Czopka et al., 2010). Tenascin-C also attenuates the phosphorylation of Akt, a crucial factor in OL survival and myelination (Czopka et al., 2010). The recent study of *in vivo* and *ex vivo* experiments for remyelination using tenascin-C or -R knockout mice displayed the promoted myelination after the treatments with cuprizone and lysolecithin (Bauch et al., 2022; Bauch and Faissner, 2022). This result indicates that tenascin-R is also a negative regulator during remyelination, as well as tenascin-C. These activities of tenascins may be useful for the application to control OL differentiation and be one of targets for the treatment of demyelinating diseases.

### Teneurin-4

Teneurins are a type of transmembrane proteins that possess the sequence homology with tenascins through tenascin-type EGF-like repeats in their large extracellular domain (Tucker and Chiquet-Ehrismann, 2006). The deficiency of teneurin-4, one of 4 teneurins in vertebrates, causes severe hypomyelination of small diameter axons in the CNS due to the reduced differentiation of OLs (Suzuki et al., 2012). The teneurin-4 homophilic binding or its heterophilic binding to the other teneurin family member promotes OL differentiation on axon-mimicking nanofibers (Hayashi et al., 2020a) (Figure 1). Moreover, teneurin-4 is specifically required for the development of type I/II OLs responsible for myelination of small diameter axons (Hayashi et al., 2020b). Interestingly, the large extracellular domain of teneurin-4 is possibly cleaved and deposited to ECM (Kenzelmann-Broz et al., 2010). Teneurin-4 may play a role in CNS myelination as not only a transmembrane protein but also an ECM protein.

### Fibronectin

Fibronectin is a cell adhesive protein in ECM and plasma. Fibronectin is composed of the typical module structure, in which type I-III modules are tandemly repeated. The major cellular receptors for fibronectin are integrins α5β1 and αvβ3 and HSPGs, including syndecans (Pankov and Yamada, 2002). In the CNS, fibronectin is expressed from astrocytes, microglia, and endothelial cells. Fibronectin negatively regulates OL differentiation (Lafrenaye and Fuss, 2010). Several studies revealed the balances between the positive and negative regulations by laminins and fibronectin (Buttery and ffrench-Constant, 1999; Lafrenaye and Fuss, 2010; Baron et al., 2014).

In the damaged CNS tissue, such as plaques in MS, the cellular fibronectin from the CNS cells is upregulated and plasma fibronectin is also detectable because of the destruction of blood-brain barrier (Sobel and Mitchell, 1989; Stoffels et al., 2013). To the contrary, the expression level of fibronectin is decreased at the remyelination process (Stoffels et al., 2013). Similar expression pattern of fibronectin is observed in experimental autoimmune encephalomyelitis (EAE) mice. The aggregation of fibronectin is formed by the reactive astrocytes due to inflammation response, while toxin-induced demyelination, a secondary inflammatory response, does not promote the formation of the aggregation (Stoffels et al., 2013; Stoffels et al., 2015). The aggregation of fibronectin inhibits myelination and remyelination since it interferes OL differentiation (Figure 1). Further, under the concept that a removal of the fibronectin aggregation may serve as a treatment for demyelinating diseases, the experiment of induced cleavage of fibronectin by MMP7 was carried out. However, the cleavage did not promote remyelination (Wang et al., 2018). These results suggest that the cleavage of fibronectin by MMP7 is not a simple solution to remove the inhibitory element from fibronectin for remyelination, possibly due to the ineffective degradation and the lack of phagocytic clearance. Further analyses are required for the application to diminish the negative activity of fibronectin in OL differentiation and remyelination.

### Hyaluronan

Hyaluronan, one of glycosaminoglycans (GAGs), composed of the straight chain structure with the repeated unit of *N*-acetylglucosamine and glucuronic acid, modulates the ECM and pericellular environments in various tissues. Hyaluronidases, Hyal-1, Hyal-2, Hyal-3, and Spam-1, degrade hyaluronan from high to low in its molecular weight and regulate the activity of hyaluronan (Sloane et al., 2010). In addition, another hyaluronidase HYBID (hyaluronan-binding protein involved in hyaluronan depolymerization), which is originally identified in the immortal renal cell carcinoma cells, is highly expressed in the brain (Michishita et al., 2006; Yoshino et al., 2017). Cell surface CD44 binds to hyaluronan and functions as its receptor (Back et al., 2005).

The activity of hyaluronan in OL differentiation and myelination has been analyzed in the pathological conditions. High molecular weight form of hyaluronan is accumulated in demyelination lesions of MS and vanishing white matter disease (Back et al., 2005; Bugiani et al., 2013). Similar accumulation of hyaluronan is observed in EAE mice (Back et al., 2005). The accumulation of high molecular weight hyaluronan, which is produced by astrocytes, inhibits remyelination in the lesions and the differentiation of OPCs to OLs (Rauch et al., 2005). In addition, it increases CD44 expression in both OL lineage cells and astrocytes (Back et al., 2005) (Figure 1). Also, the inhibitory effect of hyaluronan on OL differentiation requires the signaling of TLR2, which is highly expressed in tissues of MS patients (Sloane et al., 2010). Downstream of TLR2, MyD88 is expressed and blocks the differentiation of OLs and remyelination in MS (Decker and ffrench-Constant, 2004) (Figure 1). However, the inhibition of OL differentiation is not mediated by TLR2 signaling in vanishing white matter disease (Bugiani et al., 2013). Instead, the expression of hyaluronan synthase 2 and hyaluronidase 2, which is not detectable in MS patients, is increased in the tissue of vanishing white matter disease (Bugiani et al., 2013). From these observations, hyaluronan negatively regulates remyelination through the different mechanisms between these two diseases. Furthermore, the expression of HYBID by activated astrocytes and the degradation of hyaluronan are detected in the focal loci of the damaged tissue in EAE mice and an MS patient (Marella et al., 2018). It is possible that regulating HYBID activity is a way to control the progression of damaging tissues in MS.

### Chondroitin sulfate proteoglycans

CSPGs are another modulator of the microenvironment of ECM and pericellular regions in various tissues. CSPGs consist of a core protein and CS chains as GAGs. There are four subgroups of CSPGs: first group: lecticans including aggrecan, versican, neurocan, and brevican; second group: phosphacan/receptor-type protein-tyrosine phosphatase β; third group: small leucine-rich proteoglycans such as decorin and biglycan; fourth group: neuroglycan-C and NG2 (Galtrey and Fawcett, 2007). The CSPGs localized in ECM are mainly aggrecan, versican, neurocan, and brevican. In the CNS, CSPGs inhibit migration of OPCs and differentiation of OPCs to OLs, as well as myelination by OLs, but not proliferation of OPCs (Pendleton et al., 2013; Sun et al., 2017). Particularly, CSPGs are a mechanical and chemical barrier for the OPC migration (Sun et al., 2017). Further, the processes of OPCs treated with CSPGs, aggrecan, neurocan, and NG2, are shorten and sparse (Pendleton et al., 2013). CSPGs also have an antagonistic function to laminins, resulting from reducing the expression level of integrin β1 (Sun et al., 2017) (Figure 1). In the pathological conditions, the upregulation of CSPGs and the inhibition of OPC differentiation by CSPGs are observed at the remyelination stage in MS and animal models of MS (Lau et al., 2012; Keough et al., 2016). In the cerebral white matter injury, which is a risk for the premature of children and low birth weight, the principal component of glial scars in the white matter is CSPGs that lead to the death of OPCs (Sun et al., 2017). This inhibitory effect of CSPGs is removed by the digestion with chondroitinase ABC and the CSPG synthesis inhibitor (Lau et al., 2012; Pendleton et al., 2013; Keough et al., 2016). These discoveries may shed the light on the treatment of the diseases.

## Conclusion

In all of the tissues in our body, containing the CNS, ECM creates the microenvironment that critically controls the cellular fate. OPCs and OLs are also not the exception to be fated. Here, we have focused and discussed the major ECM proteins, however, there are many other proteins in ECM, which have not yet been characterized in OL development and myelination. In addition, ECM proteins are proteolytically processed and exert their various activities. Therefore, the functions of ECM proteins are diverse, depending on developmental stages and pathological conditions. In this sense, the comprehensive studies of ECM molecules, including the uncharacterized proteins and proteolytic fragments, will facilitate to better understand the molecular mechanisms of OL biology and be useful for the development of new agents for the diagnosis and therapy for the demyelinating and dysmyelinating diseases, as a future direction in this field.
